# The Common Oceanographer: Crowdsourcing the Collection of Oceanographic Data

**DOI:** 10.1371/journal.pbio.1001947

**Published:** 2014-09-09

**Authors:** Federico M. Lauro, Svend Jacob Senstius, Jay Cullen, Russell Neches, Rachelle M. Jensen, Mark V. Brown, Aaron E. Darling, Michael Givskov, Diane McDougald, Ron Hoeke, Martin Ostrowski, Gayle K. Philip, Ian T. Paulsen, Joseph J. Grzymski

**Affiliations:** 1 School of Biotechnology and Biomolecular Sciences, The University of New South Wales, Sydney, New South Wales, Australia; 2 Singapore Centre on Environmental Life Sciences Engineering (SCELSE), Nanyang Technological University, Singapore; 3 Department of Transport, Technical University of Denmark, Copenhagen, Denmark; 4 School of Earth and Ocean Sciences, University of Victoria, Victoria, British Columbia, Canada; 5 Genome Center, University of California, Davis, California, United States of America; 6 The ithree institute, University of Technology Sydney, Ultimo, New South Wales, Australia; 7 Costerton Biofilm Center, Department of International Health, Immunology, and Microbiology, Faculty of Health and Medical Sciences, University of Copenhagen, Copenhagen, Denmark; 8 Centre for Marine Bio-Innovation, University of New South Wales, Sydney, New South Wales, Australia; 9 Centre for Australian Climate and Weather Research, CSIRO, Aspendale, Victoria, Australia; 10 Department of Chemistry and Biomolecular Sciences, Macquarie University, Sydney, New South Wales, Australia; 11 VLSCI Life Sciences Computation Centre, University of Melbourne, Melbourne, Victoria, Australia; 12 Division of Earth and Ecosystem Sciences, Desert Research Institute, Reno, Nevada, United States of America

We live on a vast, underexplored planet that is largely ocean. Despite modern technology, Global Positioning System (GPS) navigation, and advanced engineering of ocean vessels, the ocean is unforgiving, especially in rough weather. Coastal ocean navigation, with risks of running aground and inconsistent weather and sea patterns, can also be challenging and hazardous. In 2012, more than 100 international incidents of ships sinking, foundering, grounding, or being lost at sea were reported (http://en.wikipedia.org/wiki/List_of_shipwrecks_in_2012). Even a modern jetliner can disappear in the ocean with little or no trace [1], and the current costs and uncertainty associated with search and rescue make the prospects of finding an object in the middle of the ocean daunting [2].

Notwithstanding satellite constellations, autonomous vehicles, and more than 300 research vessels worldwide (www.wikipedia.org/wiki/List_of_research_vessels_by_country), we lack fundamental data relating to our oceans. These missing data hamper our ability to make basic predictions about ocean weather, narrow the trajectories of floating objects, or estimate the impact of ocean acidification and other physical, biological, and chemical characteristics of the world's oceans. To cope with this problem, scientists make probabilistic inferences by synthesizing models with incomplete data. Probabilistic modeling works well for certain questions of interest to the scientific community, but it is difficult to extract unambiguous policy recommendations from this approach. The models can answer important questions about trends and tendencies among large numbers of events but often cannot offer much insight into specific events. For example, probabilistic models can tell us with some precision the extent to which storm activity will be intensified by global climate change but cannot yet attribute the severity of a particular storm to climate change. Probabilistic modeling can provide important insights into the global traffic patterns of floating debris but is not of much help to search-and-rescue personnel struggling to learn the likely trajectory of a particular piece of debris left by a wreck.

Oceanographic data are incomplete because it is financially and logistically impractical to sample everywhere. Scientists typically sample over time, floating with the currents and observing their temporal evolution (the Langrangian approach), or they sample across space to cover a gradient of conditions—such as temperature or nutrients (the Eulerian approach). These observational paradigms have various strengths and weaknesses, but their fundamental weakness is cost. A modern ocean research vessel typically costs more than US$30,000 per day to operate—excluding the full cost of scientists, engineers, and the cost of the research itself. Even an aggressive expansion of oceanographic research budgets would not do much to improve the precision of our probabilistic models, let alone to quickly and more accurately locate missing objects in the huge, moving, three-dimensional seascape. Emerging autonomous technologies such as underwater gliders and in situ biological samplers (e.g., environmental sample processors) help fill gaps but are cost prohibitive to scale up. Similarly, drifters (e.g., the highly successful Argo floats program) have proven very useful for better defining currents, but unless retrieved after their operational lifetime, they become floating trash, adding to a growing problem.

Long-term sampling efforts such as the continuous plankton recorder in the North Sea and North Atlantic [3] provide valuable data on decadal trends and leveraged English Channel ferries to accomplish much of the sampling. Modernizing and expanding this approach is a goal of citizen science initiatives. How do we leverage cost-effective technologies and economies of scale given shrinking federal research budgets?

## Citizen Scientists Can Make an Important Contribution

There are many actions that can be taken to improve the precision of our models, but the most obvious is to increase spatial and temporal density of our observations. However, the cost of oceanographic research vessels makes this impractical. The inevitable conclusion is that observations must be obtained by some other means. We propose a worldwide effort to empower sailors and retrofit sailboats to increase coverage of sample and data collection along common routes around the world.

Modern oceanographic research vessels are large and expensive because they are designed to be general-purpose scientific platforms. They are sophisticated laboratory facilities that serve the diverse needs of the scientific community for many decades. These vessels are costly because their scientific capabilities are both wide ranging and deep penetrating. The ocean is too vast for any vessel to see very much of it, no matter its capabilities. Maximizing the number of observers, rather than the capabilities of observers, requires a very different approach to the choice of vessel, personnel, instrumentation, and protocol.

Can meaningful data be collected with the kind of narrowly focused, low-cost instrumentation that is easily mass produced and deployable? If so, what vessels will carry it, and what personnel will operate it? Many aspects of modern oceanography, such as locating an underwater object, require sophisticated equipment and trained experts. However, some of the most important types of observations require only that one be in the right place at the right time with simple instrumentation or sampling equipment. Important data can be gathered by anyone who can follow basic instructions. This is the premise of “citizen science” (Box 1). Rather than dispatching scientists into the environment to collect data, scientists may instead train people who already interact with the environment to apply the scientific method to phenomena they already observe. With or without an invitation, citizen scientists exist. There is an urgent need to make a place for them in the scientific community.

**Box 1. Citizen Science Primer**

Citizen science is a manner of collecting data and observations in which collaborators who may lack credentials and formal institutional affiliation can contribute to the work. Because one does not vet collaborators on the basis of affiliation and credentials, a citizen science research project must be specific and self-contained in terms of what is asked of collaborators.

For example, rather than requiring a master's degree in entomology, a citizen science project might ask if a candidate can learn to identify a particular species of ant using a dichotomous key.

## Historical Perspective

A mistaken and modern perception is that science is an elitist profession, relegated to well-funded laboratories with complex instrumentation run by professors with years of advanced education. Historically, this was not always the case. In fact, people who conducted scientific research as a hobby achieved some of the greatest discoveries in history. For example, Leonardo da Vinci painted portraits for income while doing science in his spare time. Gregor Mendel, an Augustinian friar, discovered the basis of genetic inheritance while working in the garden of his monastery, and Michael Faraday laid the foundation of electromagnetic induction while working as an apprentice bookbinder and bookseller, educating himself. Arguably the most famous “citizen oceanographer” was Charles Darwin, who had no formal training in biology but became one of the most celebrated and influential evolutionary biologists in history. He detailed the geology and formation of coral reefs during the 1832–1836 voyage of HMS *Beagle*. Similarly, Benjamin Franklin, also a “citizen oceanographer,” published a number of ideas on Atlantic Ocean currents, catamaran hulls, and sea anchors and designed a spill-proof bowl for eating soup on board a ship in stormy seas.

Despite this long tradition, the involvement of amateurs in oceanographic discoveries declined in the 20th century, perhaps contributing to the growing misunderstanding of scientific jargon by the public when it pertains to ocean and atmospheric circulation. This has led to the current political shape-shifting of scientific results (e.g., the climate change debate). In the last few years, mostly due to technological breakthroughs, we have witnessed revitalization in the participation of civilians in data collection: Alan Irwin described the social aspects of this revolution in 1995 [4] and coined the term “citizen science.”

The United States National Weather Service Cooperative Observer Program (NWS-COOP) is a great example of a successful citizen science initiative. The program was established in 1890 and utilizes a network of more than 11,000 volunteers to provide observational data of basic weather parameters. Similarly, the USA National Phenology Network is a group of scientists and trained citizen scientists who collect observations about plant and animal phenology. This multidecadal program helps scientists, for example, better understand climate change [5].

With thoughtfully designed and well-tested equipment and protocols, citizen scientists can gather vast quantities of oceanic data or samples for analysis (Box 2). Three technologies have provided the technical means for networked data collection: the miniaturization of sample collection devices, the progressive reduction in the cost of sequencing, and the computing and easy data sharing of cloud-based analysis [6].

**Box 2. Citizen Scientists Could Collect Several Types of Important Oceanographic Data**

- *Biological samples*. Our ability to monitor the status of the world's oceans and evaluate the effect of human activities depends on quantifying the microbial communities in all ocean basins and understanding their community structure and function in response to natural and anthropogenic perturbations. Marine microbes are the foundation of the planet's trophic networks and play a critical role in planetary biogeochemical processes. They are the sentinels of the sea and respond rapidly to perturbations (e.g., Deepwater Horizon [22]).
- *Basic physical parameters*. Temperature and conductivity, coupled with depth, reveal the hidden structure of ocean currents. Much of these structures cannot be directly observed with satellite and other remote-sensing technologies.
- *Surface weather conditions*. Most of the world's oceans are not covered by the sophisticated Doppler radar systems used in terrestrial weather forecasting. Weather satellites can reveal a great deal by observing clouds from above but have limited ability to directly observe conditions underneath them. Simple rainfall observations and observations of sea surface and wave heights would be helpful for modeling.
- *Debris sightings*. The abundance and trajectory of debris in the ocean have a large impact on marine ecosystems. When it is possible to track debris, it also can be used to monitor the evolution and status of ocean currents.

Oceanography using large ships has a significant carbon and economic footprint, but spatially extensive and temporally intensive data are needed. More specifically, in marine microbiology, data collection has become the bottleneck, since it is currently impossible to quantify the totality of oceanic microbial communities and their environmental drivers by remote sensing or individual research cruises. Expansive budgetary cuts to environmental sciences around the globe and the concurrent need to renew an aging fleet of ocean vessels [7] underscore the urgency.

## Long-Term Oceanographic Research with Citizen Scientists

How do we increase temporal and spatial coverage of the ocean? A single oceanographic research vessel can cover a small fraction of the ocean. During a continuous year at sea, without stopping for fuel or crew change, a single research vessel could cover ∼3% of ocean-area sampling at a distance of 1 degree, traveling at 10 knots between stations and only stopping at each station for two hours. Despite this being an enormous area, representing 9.7 million km^2^ (area calculation from the Goode Homolosine projection), it conservatively would cost at least US$15 million for the boat, the crew, the science, and the scientists. Twenty vessels would be required to operate at this intensity to cover the midlatitude region, putting into perspective the costs of conducting oceanography on a global ocean-basin scale. The carbon footprint of this effort would be enormous. The costs would exceed US$300 million dollars, and the results would still exclude the entire high-latitude oceans.

**Figure 1:**
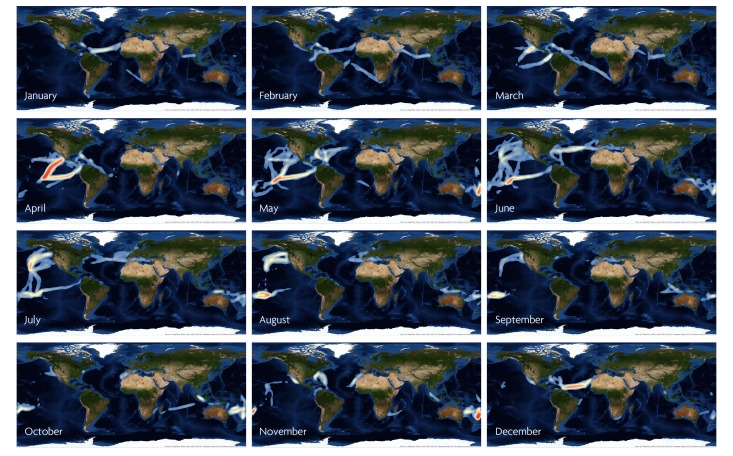
Month-by-month maps of sailing yacht transits around the world. Data are collected from the YOTREPS network (http://www.pangolin.co.nz/) of cruising yachts worldwide and plotted with Esri ArcGIS 10.2.1. Density of yacht traffic is highest in red. Note the seasonal patterns of transits in the various oceans: high density of traffic during the “Coconut Milk Run” in the Pacific beginning in April, eastward Atlantic ocean traffic during the boreal summer and westward during the boreal winter, and passages to Alaska in the heart of the boreal summer. Used with permission. Esri, DigitalGlobe, 2014.

Citizen oceanography, specifically science conducted aboard sailing yachts, would overcome many of these hurdles and empower civilian scientists with the pride of data contribution to science, providing an incredible opportunity for outreach as well as improving science education and increasing public awareness. Participation of a small fraction of the thousands of vessels that continuously cruise remote parts of the oceans (Figure 1) could comprise a global oceanographic monitoring network that would boost the predictive power of scientific models. This would be a natural group of citizen scientists inherently motivated by their love of sailing and empirical knowledge of the beauty, power, and vastness of the world's oceans. Given the complexities and importance of the coastal ocean, an obvious question is, why not include coastal recreational sailors in this call to action? The coastal waters present problems related to permitting, and deregulated sampling would infringe on the exclusive economic rights of nations' territorial waters. However, other initiatives such as Ocean Sampling Day [8] can be extended to cover multiple days throughout the year. In partnership with coastal schools and universities, this is a huge pool of citizen scientists and citizen data collectors.

As a specific example, we know relatively little about the inventory of microorganisms and their variability in the oceans. Biological sampling by citizen scientists could be accomplished using mass-produced Niskin bottles, preloaded with fixatives, for flow cytometry and nutrient analysis. A basic conductivity-temperature-depth (CTD) device could be manufactured using standard mass-market consumer electronic practices for measuring physical properties. Inexpensive digital weather stations already exist for recording conditions and are commonplace on small, private vessels. A wide-angle camera and an inexpensive embedded computer could be used to automatically identify and catalogue debris with image-processing software.

Repetitive sampling through time is important to grasp the complexities of dynamic systems (cf. [9]–[11]). The long-term ecological research (LTER) model for understanding ecosystem function and change across relevant temporal scales has produced numerous significant observations [12],[13]. Two important long-term research observatories in the Atlantic (Bermuda Atlantic Time-series Study [BATS]) and Pacific (Hawaiian Ocean Time-series [HOT]) have revolutionized our understanding of biology and chemistry in the ocean [14]. Yet, these are only two stations in the middle of vast oceans.

There is repetitive, seasonal yacht traffic along common sailing routes around the globe (Figure 1). At least 5,000 sailing yachts travel the oceans every year using several popular routes. For example, ∼400 yachts per year embark on the Pacific Coconut Milk Run, which is approximately a 6,000 nautical mile journey from the western US or Panama Canal to New Caledonia in the Southwest Pacific. This popular route is marked by relatively mild weather and easterly trade winds. The Atlantic Ocean has similar seasonal and favorable sailing routes. The trade wind route (westward from Canaries to the Caribbean) is mostly sailed in winter, while the Bermuda-Azores (eastward) is popular in the summer (Figure 1). Both attract hundreds of sailors every year, as they are a part of every sailor's “wish list,” having been used since the Age of Discovery in the early 15th century. If a metadata and sample collection system were in place, sailors on popular routes such as these could be a part of a larger, LTER-like sampling collective. Ocean-going sailors have an extensive network already in place for communication, passage notes, weather, etc. (e.g., http://www.cruiserswiki.org/; http://pangolin.co.nz/); expanding this infrastructure is trivial compared to current research vessel costs. These routes can be used to monitor the ocean repetitively for interannual and long-term changes, much like the NWS-COOP or National Phenology Network programs.

Any data gaps in areas with lower density of recreational sailors (e.g., high latitudes) could be filled by engaging commercial shipping companies, which have vessels that operate in those waters [15].

## Lessons Learned from the Indigo V Indian Ocean Expedition

Many projects have struggled with the unique technical, logistical, organizational, and ethical issues that arise for each discipline when researchers endeavor to involve citizen scientists. In 2013, the Indigo V Indian Ocean Expedition was conceived as a pilot project and learning laboratory for citizen science approaches to oceanography. The team sailed S/Y *Indigo V* —a 61-foot Nautor Swan sailing yacht—across the Indian Ocean from Cape Town, South Africa, to Phuket, Thailand. The three legs of the journey covered approximately 5,800 nautical miles. During this expedition, instruments and methods adaptable to citizen scientist deployment were tested aboard small vessels not designed or equipped for research. In all but the heaviest seas, the crew was able to inventory the surface water population of bacterioplankton using a simple pump and filtration apparatus and make basic measurements of ocean physics and chemistry. DNA and RNA were successfully recovered from samples preserved using a nontoxic salt solution (RNAlater, Qiagen, Valencia, California).

The prototype ocean sampling microbial observatory (OSMO) is currently being ruggedized and automated for citizen-science-based collections of bacterioplankton samples. This device is being developed as a collaboration between the *Indigo V* team members in their laboratories in the US, Singapore, and Australia and will autonomously sample microbial populations onto filters and preserve them. The sailor/scientist would be responsible for metadata collection, uploading that data to a central database, and shipping the samples back to the lab for processing. The total cost of design, prototyping, field-testing, and commercializing this device is less than US$200,000. Ultimately, the total cost of microbial sample collection, processing, and sequencing using this approach and device could be reduced to ∼US$1,500 per sample or less. It will be cost-effective to inventory the microbial community of an ocean basin. The citizen science can be extended to the data analysis phase by online annotation tutorials.

Many aspects of science aboard a sailing yacht are similar to science aboard a research vessel. There is an intense focus on collecting samples; by simplifying the sample collection methodology, this task can be taught to sailors. Similarly, observations aboard a sailing yacht are a part of daily life—situational awareness is essential for the safety of the crew and the boat. Wind speed, sea state, sea height, and currents are observational data that can be collected by sailors to improve global ocean models (or verify predictions).

By employing sail power, the Indigo V project demonstrated that an entire four-month expedition, sampling a wide range of waters with a variety of instruments, costs the equivalent of a day or two of ship time aboard an oceanographic research vessel. Relative to a typical research vessel, the use of sail power reduced carbon emissions resulting from vessel operations by approximately 1000-fold. For comparison, a recent global oceanographic project (http://www.expedicionmalaspina.es/) collected samples at 180 stations with a budget of US$23 million [16],[17], and another (http://oceans.taraexpeditions.org/) sampled 375 stations with a budget of over US$12 million [18]–[21]. The Indigo V Indian Ocean Expedition collected samples at 50 stations for less than US$75,000. Imagine what the thousands of yachts that are already out on the water could do.
